# Effects on metabolic parameters in young rats born with low birth weight after exposure to a mixture of pesticides

**DOI:** 10.1038/s41598-017-18626-x

**Published:** 2018-01-10

**Authors:** Terje Svingen, Louise Ramhøj, Karen Mandrup, Sofie Christiansen, Marta Axelstad, Anne Marie Vinggaard, Ulla Hass

**Affiliations:** Division of Diet, Disease Prevention and Toxicology, National Food Institute, Technical University of Denmark, Kemitorvet Bldg. 202, Kgs. Lyngby, DK-2800 Denmark

## Abstract

Pesticide exposure during fetal life can lead to low birth weight and is commonly observed in reproductive toxicology studies. Associations have also been found in low birth weight babies born from pesticide-exposed gardeners. Since low birth weight is also linked to metabolic disorders, it can be speculated that early life exposure to pesticides could increase the risk of becoming obese or developing diabetes later in life. We have analyzed potential long-term effects of gestational and lactational exposure to a low dose mixture of six pesticides that individually can cause low birth weight: Cyromazine, MCPB, Pirimicarb, Quinoclamine, Thiram, and Ziram. Exposed male offspring, who were smaller than controls, displayed some degree of catch-up growth. Insulin and glucagon regulation was not significantly affected, and analyses of liver and pancreas did not reveal obvious histopathological effects. Efforts towards identifying potential biomarkers of metabolic disease-risk did not result in any strong candidates, albeit leptin levels were altered in exposed animals. In fat tissues, the key genes *Lep*, *Nmb* and *Nmbr* were altered in high dosed offspring, and were differentially expressed between sexes. Our results suggest that early-life exposure to pesticides may contribute to the development of metabolic disorders later in life.

## Introduction

Fetal programming is a tightly regulated, highly complex process that ensures the development of viable offspring. It largely depends on an innate program inherited from parents at conception, but it is also susceptible to influences from the external environment. The womb protects the fetus from the outside world, but it is far from impermeable. Apart from essential nutrients and oxygen provided by the mother, environmental chemicals can also find their way to the fetus. Since many chemicals can interfere with molecular and cellular signaling events, fetal programming can be disturbed and cause permanent alterations with long-lasting consequences. For instance, human studies have revealed strong associations between low birth weight and late-onset metabolic diseases such as insulin resistance and type-2 diabetes^1,2^; findings supported by many animal studies showing, among other things, that protein restriction during fetal life can lead to low birth weight, increased blood pressure and altered glucose tolerance in offspring of rats^3–5^, guinea pigs^6,7^ and sheep^8^.

There are many reasons why a child is born small, not least because of premature delivery or a multiple pregnancy. But exposure to different environmental pollutants can also negatively affect human fetal growth^9^. For example, intrauterine exposure to tobacco smoke^10^, lead^11^ or phthalates^12^ can all stunt fetal growth. The prevailing view is that the fetus responds to its immediate environment, whereby complex regulatory pathways and adaptive responses are initiated so that the fetus become best equipped for postnatal life^1^. The flipside to such adaptive responses however, is that a low birth weight also is a risk factor for developing various late-onset diseases, including obesity and type-2 diabetes^2^. A good example of this comes from a study looking at the effects of pesticide exposure in children born from mothers working in greenhouses^13^. Here, associations were found between high pesticide exposure during early pregnancy and low birth weight, but it was also found that the high-exposure, low birth weight children had a stronger propensity for increase in Body Mass Index (BMI) towards school age. More subtle associations were also observed between high exposure and potential biomarkers of metabolic disease, insulin-like growth factor 1 (IGF-1) and thyroid-stimulating hormone (TSH)^13^. Low birth weight is also frequently observed in animal studies examining the effects of *in utero* exposure to different chemicals, including pesticides, and it is a clear sign of developmental toxicity and a warning flag that the pesticide can disrupt fetal development or growth, both in animals and humans.

We recently reported on the combined low-dose effect of six currently used pesticides, showing a dose-dependent decrease of birth weight in rat offspring whose mothers had been exposed orally from gestational day 7 and throughout the rest of gestation^14^. The pesticide doses were singularly below No Observed Adverse Effect Levels (NOAELs) for decreased birth weight and the effect-outcome was driven by the combined action of more than one pesticide. These results, together with other observations linking low birth weight with fetal exposure to environmental chemicals^9,13^, encouraged us to investigate whether the rat offspring from our study would develop signs of metabolic syndrome as they grew older, but also to potentially identify early biomarkers for late onset metabolic diseases.

## Results

### Effects on body and organ weights following early life exposure to a low-dose mixture of pesticides

We previously reported a lower birth weight in rat offspring following exposure to the low dose mixture of six pesticides administered from gestational day 7 until pup day 16 (staged relative to day of conception)^14^. Both male and female rats were significantly smaller at pup day16 in the highest exposure group, but the effect was no longer significant at 5–6 months of age. As summarized in Table 1, this was also the case for livers and retroperitoneal fat pads. Some significant effects were observed in exposed animals on pup day 16, with relative weights of livers and retroperitoneal fat pads being greater in the pup day 16 rats at high doses, but not at 5–6 months of age. No significant effects on femoral muscle weights were observed. Notably, a large variation in liver weights was seen in the Mix-5% group at 16 days of age, especially in males (coefficient of variance (CV) = 31.6%). Thus, statistical analysis on liver weights excluding data from the Mix-5% group was also performed to avoid false negative results (Table 1). Finally, histological assessment of livers showed no changes except for decreased sign of glucogen accumulation (decreased rarefaction) in exposed male livers (13 out of 19 control males and 2 out of 16 high-dose males, p = 0.002).

**Table 1:** Body and organ weights of rat offspring at pup day 16 and 5–6 months of age. Absolute organ weights were analyzed by ANOVA with body weight as a covariate and Dunnett’s test with LSMeans to determine differences between treated groups and controls. Body weights and relative organ weights were analyzed by ANOVA and Dunnett’s post hoc test with LSMeans. Mean ± SD. *p < 0.05. ^#^p < 0.05 when Mix-5% is excluded from the statistical analysis of livers in pup day 16 animals.

Offspring; pup day 16	Control	Mix-5%	Mix-16%	Mix-37.5%
	n = 18	n = 17	n = 17	n = 18
***Males–absolute weight (g)***				
Body weight	34 ± 5.3	30 ± 5.2	32 ± 5.4	29 ± 5.4*
Retroperitoneal fat pad	0.062 ± 0.020	0.055 ± 0.015	0.065 ± 0.018	0.068 ± 0.021*
Liver	0.870 ± 0.148	0.819 ± 0.223*	0.843 ± 0.149^#^	0.793 ± 0.146^#^
Femoral muscle	0.060 ± 0.020	0.052 ± 0.015	0.052 ± 0.018	0.048 ± 0.018
***Males–relative weight (g)***				
Retroperitoneal fat pad	0.181 ± 0.045	0.186 ± 0.047	0.205 ± 0.039	0.230 ± 0.053*
Liver	2.557 ± 0.115	2.827 ± 0.893	2.657 ± 0.104^#^	2.732 ± 0.154^#^
Femoral muscle	0.179 ± 0.064	0.176 ± 0.051	0.165 ± 0.048	0.165 ± 0.055
	**n = 18**	**n = 19**	**n = 14**	**n = 17**
***Females–absolute weight (g)***				
Body weight	33 ± 5.0	32 ± 7.1	29 ± 4.0	29 ± 4.9*
Retroperitoneal fat pad	0.045 ± 0.017	0.049 ± 0.014	0.036 ± 0.008	0.046 ± 0.012*
Liver	0.869 ± 0.140	0.786 ± 0.191	0.774 ± 0.112	0.803 ± 0.148^#^
Femoral muscle	0.053 ± 0.022	0.057 ± 0.026	0.046 ± 0.014	0.054 ± 0.018
***Females–relative weight (g)***				
Retroperitoneal fat pad	0.132 ± 0.041	0.156 ± 0.025	0.125 ± 0.021	0.160 ± 0.034*
Liver	2.592 ± 0.103	2.532 ± 0.427	2.657 ± 0.117	2.780 ± 0.115
Femoral muscle	0.161 ± 0.072	0.181 ± 0.072	0.160 ± 0.059	0.191 ± 0.072
**Offspring; 5–6 months**	**Control**	**Mix-5%**	**Mix-16%**	**Mix-37.5%**
	**n = 18**	**n = 19**	**n = 14**	**n = 17**
***Males–absolute weight (g)***				
Body weight	441 ± 42.2	421 ± 49.2	411 ± 47.9	410 ± 26.1
Retroperitoneal fat pad	4.425 ± 1.799	4.100 ± 1.544	4.668 ± 1.607	4.677 ± 1.522
Liver	12.08 ± 1.06	11.66 ± 2.15	11.48 ± 1.20	11.43 ± 1.08
***Males–relative weight (g)***				
Retroperitoneal fat pad	0.995 ± 0.347	0.961 ± 0.316	1.118 ± 0.320	1.139 ± 0.343
Liver	2.592 ± 0.103	2.532 ± 0.427	2.657 ± 0.117	2.780 ± 0.115
	**n = 18**	**n = 19**	**n = 14**	**n = 17**
***Females–absolute weight (g)***				
Body weight	242 ± 24.4	234 ± 18.5	232 ± 16.5	228 ± 16.8
Retroperitoneal fat pad	1.486 ± 0.365	1.521 ± 0.392	1.575 ± 0.547	1.586 ± 0.353
Liver	6.570 ± 0.949	6.375 ± 0.669	6.434 ± 0.706	6.209 ± 0.560
***Females–relative weight (g)***				
Retroperitoneal fat pad	0.611 ± 0.120	0.648 ± 0.150	0.672 ± 0.195	0.697 ± 0.153
Liver	2.712 ± 0.239	2.727 ± 0.156	2.774 ± 0.271	2.728 ± 0.167

**Figure 1:**
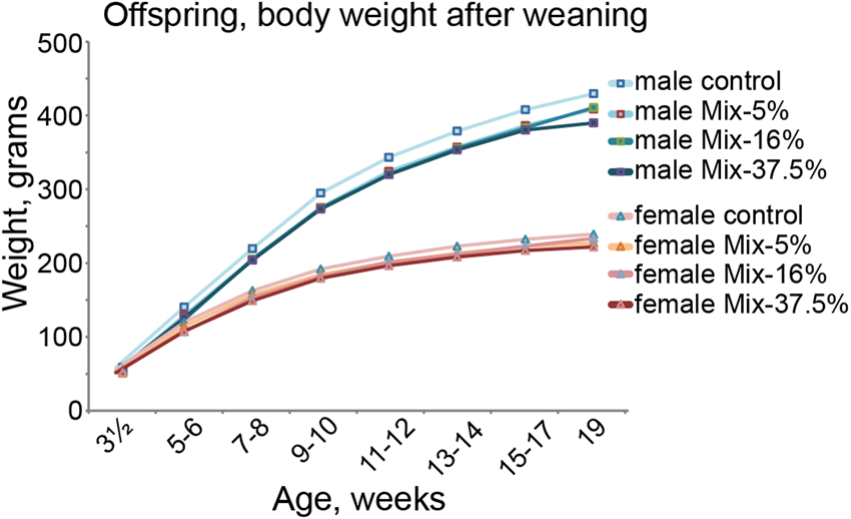
Offspring body weight after weaning, male and female. Weights were obtained at different ages for the different blocks and grouped according to the age of the offspring for the purpose of depicting data. The statistical analysis was performed with the exact timing of the weight included. For males, the mean weight in all exposure groups was significantly reduced compared to controls. n = 18–20. Group means are depicted.

Body weight was also monitored over time to observe potential trends in catch-up growth (Fig. 1). For male offspring, the average body weight was lower in all dose-groups compared to control (Mix-5%, p = 0.0172; Mix-16%, p = 0.0391; Mix-37.5%, p = 0.0169). For female offspring, a tendency toward lower body weights (Dunnett’s adjusted p = 0.04) were observed in the Mix-37% group. Thus, despite there being no significant difference between groups among the weaned offspring, the male offspring from the dosed groups weighed less than the controls for the whole time-period after weaning.

### Effects on metabolic parameters following early life exposure to a low-dose mixture of pesticides

**Figure 2:**
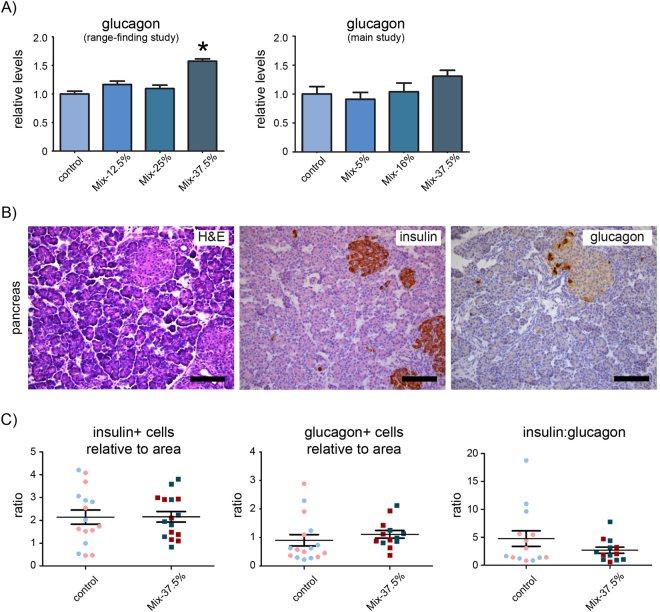
Glucagon levels and morphometric analyses of pancreatic tissue in offspring at pup day 16. (**A**) To improve comparison of data between the two studies data is presented in the form of normalized data relative to control and as mean + SEM. *p < 0.05 by ANOVA with Dunnett’s post hoc test. N = 6–9 in the dose-range-finding study and N = 17–18 in the main study. (**B**) Relative area in the pancreas occupied by β-cells (insulin positive) and α-cells (glucagon positive) were assessed by point counting. Images are from a representative pancreas (male) from a control offspring at 16 days of age stained with H&E (left) or using antibodies against insulin (middle) or glucagon (right) with slight H&E background staining. (**C**) No changes were found in the insulin/pancreas ratio (left), the glucagon/pancreas ratio (middle) or in the ratio between insulin and glucagon producing cells (right) on pup day 16. N = 13–14 per group. Scale bars = 100 µm.

In a smaller range-finding study conducted in order to optimize exposure doses, as described in^14^, glucagon levels were observed to be around 160% that of control animals in the highest dose group at pup day 16 (shown here in Fig. 2A, left). The same measurements were therefore repeated on offspring from the present study at pup day 16. The administered dose was different in the low- and mid-dosed group, but same in the high dose group (Mix-37.5%). The glucagon level again appeared to be slightly elevated (approx. 130% that of control) in the highest dose group, albeit not statistically significant (Fig. 2A, right).

We next analyzed the pancreas at pup day 16 and in adult offspring by semi-quantitative histological point counting to assess whether the area occupied by glucagon- and insulin-producing cells was altered in the highest exposed animals (Mix-37.5%). When comparing the overall ratio of the area of insulin- or glucagon-producing cells relative to the area of pancreas, no significant changes were seen between the highest dosed rats at pup day 16 compared with controls (Fig. 2C) nor in adult offspring (Data not shown). Thus, although a tendency towards a lower insulin-to-glucagon ratio was discernable, these effects were not significant at the tested dose levels.

**Figure 3:**
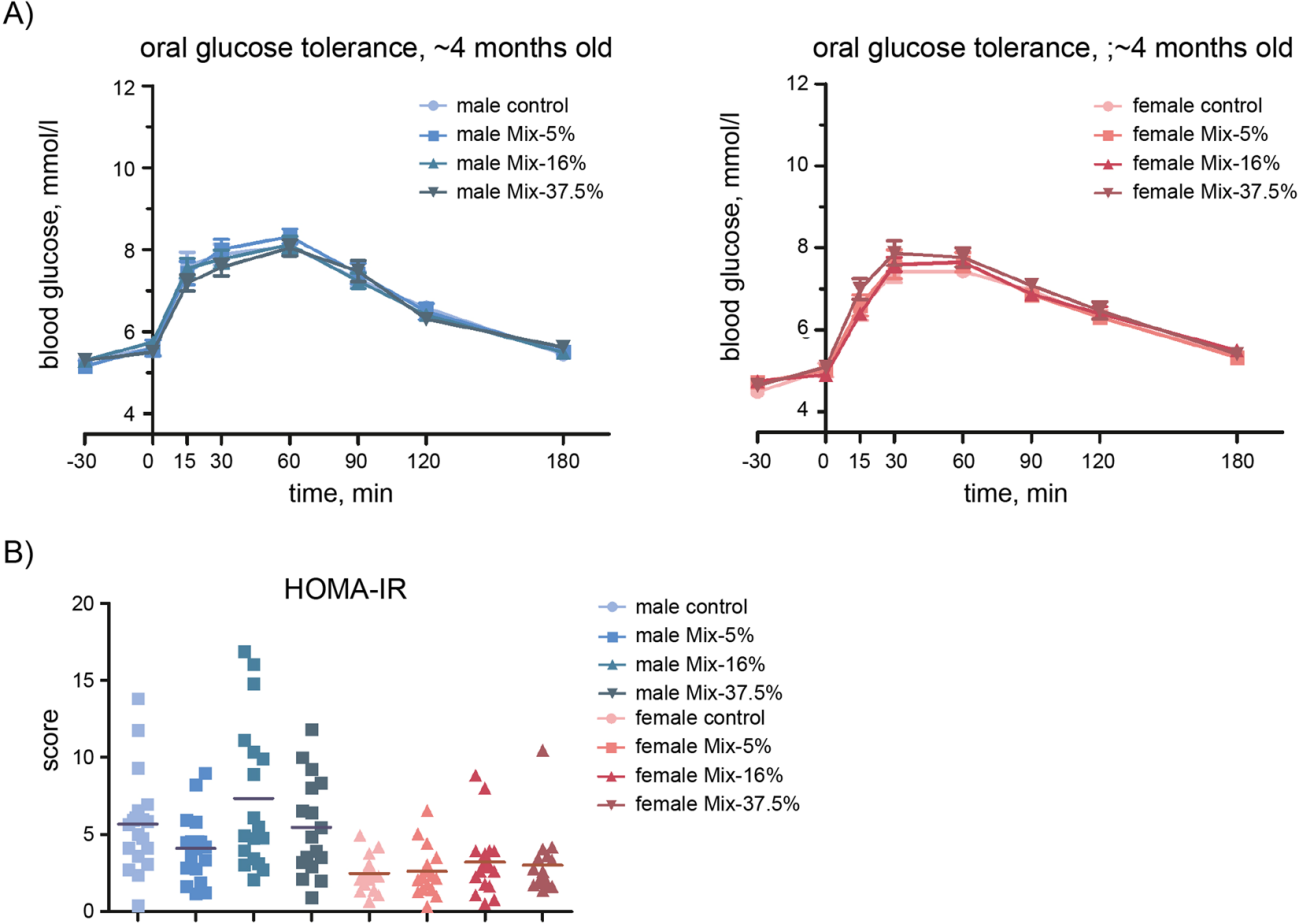
Glucose tolerance in offspring at ~4 months of age. (**A**) A standard glucose tolerance test was performed, with blood glucose levels measured at set time points starting 30 min before glucose administration and final measurement at 180 min. Males (left panel) and females (right panel) are graphed separately. Data points represents Mean + SEM; N = 17–20 each group (**B**) A homeostatic model assessment (HOMA) for insulin resistance (IR) was calculated, with each data point representing one animal. Cross bars denote mean HOMA-IR score; N = 17–20.

**Figure 4:**
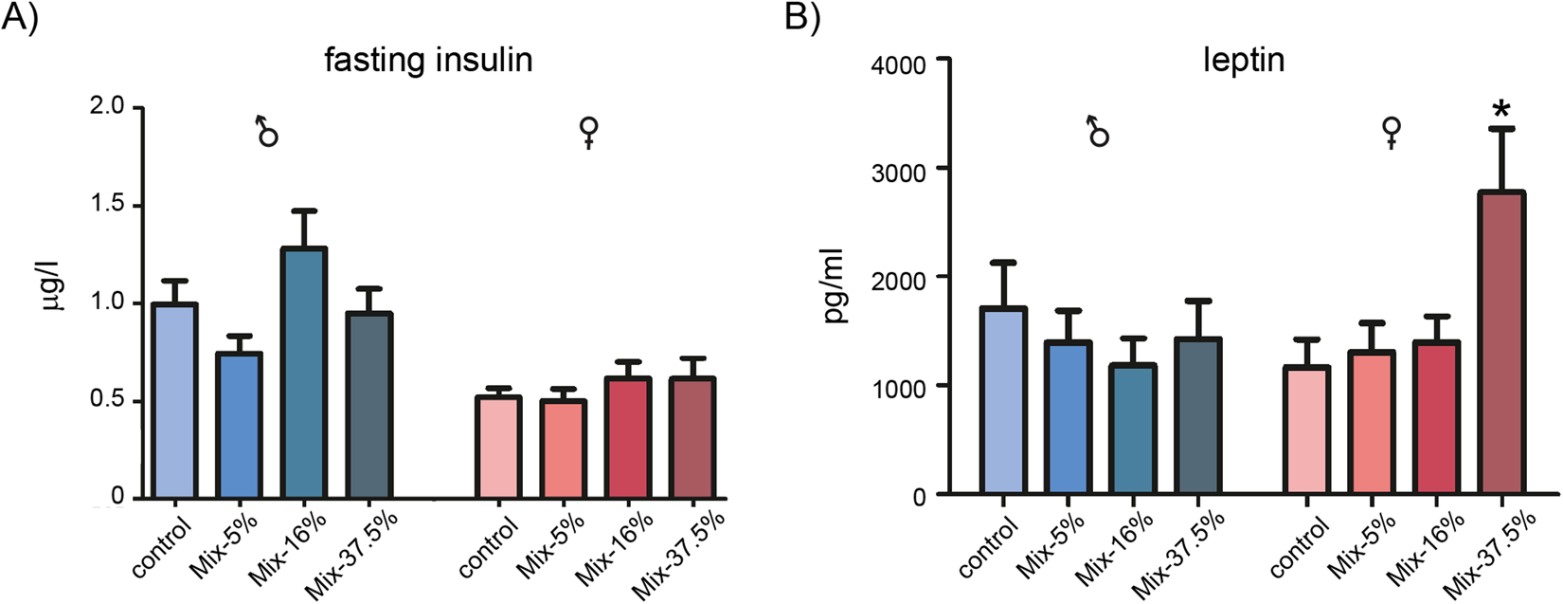
Fasting insulin and leptin levels in plasma from offspring at ~4 months of age. (**A**) N = 17–20 Mean + SEM. (**B**) Data is presented as Mean + SEM. *p < 0.05 by ANOVA with Dunnett’s post hoc test. N = 17–20 for the males and N = 10–14 for the females.

Overall, the offspring from the highest exposed groups were still smaller than controls at ~4 months of age, albeit no longer statistically significant (Table 1). In other words, the difference in overall body weight was less in the young adults than when they were of prepubertal age. This could indicate a degree of catch-up growth, so we analysed the overall food intake and energy efficiency in these older animals to potentially detect signs of metabolic disturbance. No significant change in either of the two parameters was observed in any of the exposure groups (Data not shown). An oral glucose tolerance test was performed on the animals one week after the food intake test, but no differences were observed between any groups (Fig. 3A). A homeostatic model assessment (HOMA) for insulin resistance (IR) score (HOMA-IR) was also calculated from the data, but revealed no statistically significant changes between the control group and treated animals (Fig. 3B). We also measured plasma insulin and leptin levels. For insulin we saw no treatment-related effect (Fig. 4A). For leptin, however, we observed a significantly elevated plasma level in the highest dosed female offspring (Fig. 4B).

### Gene expression profiling of fat tissue

Since leptin levels were affected in exposed female rats, we performed gene expression analyses on retroperitoneal fat pad from the 5–6 months old offspring. The aim was to see if metabolic pathways were dysregulated in young adults following early life exposure to pesticides. Initially, a targeted 84-gene array was run on retroperitoneal fat pads. Three fat samples, two male and one female, were included from control and each of the three exposure groups. Some genes were altered more than 2-fold in exposed animals relative to control. For instance, *Nmbr* and *Npy1r* were upregulated in all exposure groups, whereas *Lepr* and *Nmb* were downregulated (Suppl. Figure 1 and Suppl. File 1).

**Figure 5:**
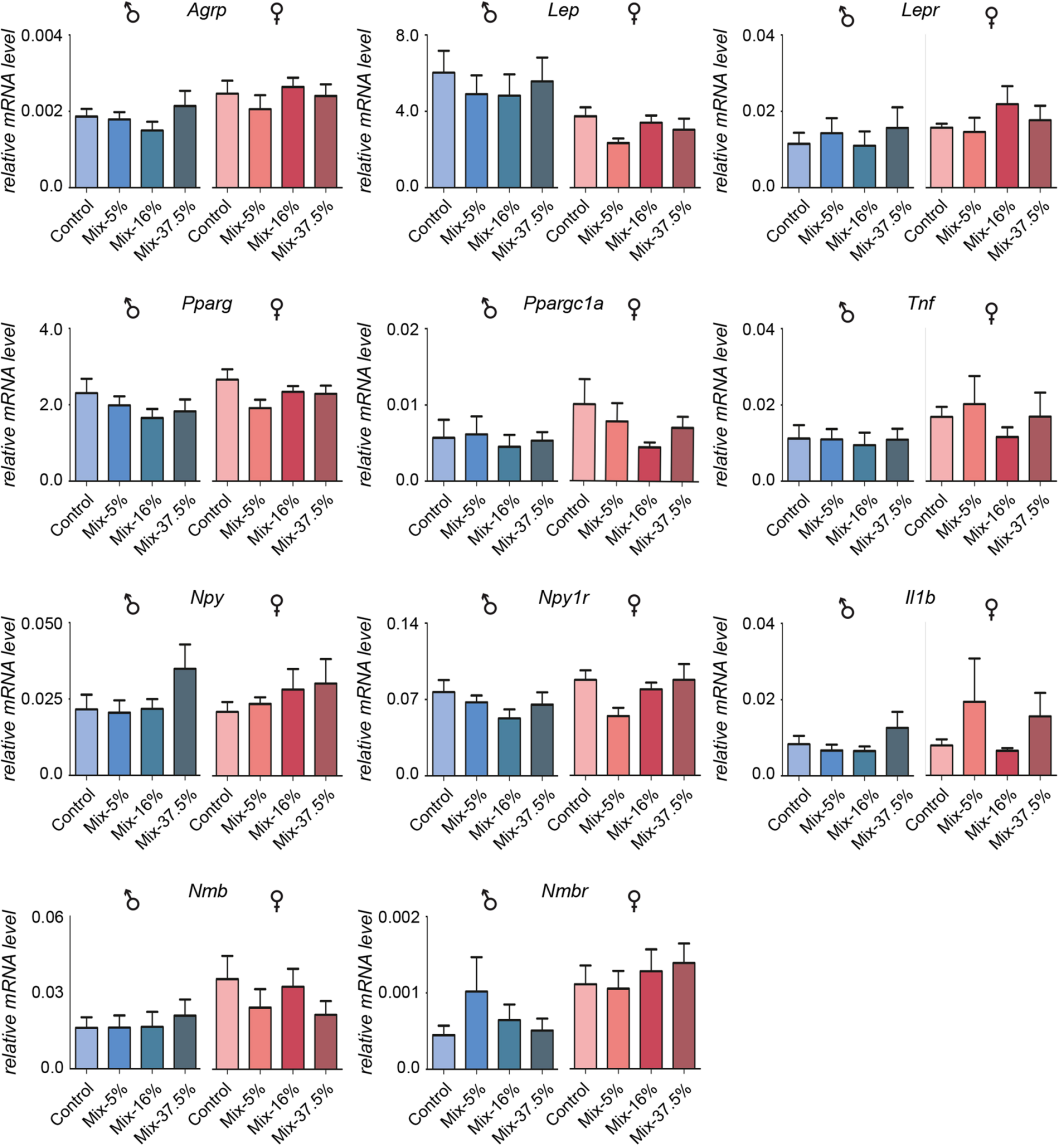
Relative mRNA expression of obesity-related genes in fat tissue in 5–6 months old offspring. The targeted gene array was verified by TaqMan RT-qPCR assays on 11 genes across the sex-specific groups (n = 8–9 from each group). Pink and blue bar represents female and male groups, respectively. Although some genes showed a trend towards either up- or down-regulation, none were significantly altered relative to controls as determined by one-way ANOVA analysis. In control animals, *Nmbr* was expressed significantly higher in females than in males (p = 0.030), with *Nmb* appearing higher in females (p = 0.075) and *Lep* appearing higher in males (p = 0.088) as determined by two-tailed, unpaired t-test analyses.

Additional RT-qPCR analyses were carried out on a larger sample size for eleven selected genes. The relative fold change in transcript abundance was less than what was observed on the array, and the trends were not upheld for all genes (Fig. 5). Genes that were either up- or down-regulated in the RT-Profiler array correlated with up- or down-regulated genes as assessed by RT-qPCR analysis. Since several genes showed a sex-specific expression pattern, data were split into male and female groups. Again, the trends were maintained but differences in mRNA levels were not statistically significant, neither for male nor female samples. Pooled data were also not statistically significant (Data not shown). A significant difference in *Nmbr* transcript abundance was observed between male and female control tissues (p = 0.030), with mRNA levels approximately 2.5-fold higher in female fat tissue. *Nmb* displayed a strong trend towards being upregulated in female fat tissue at 2.2-fold (p = 0.075), whereas *Lep* trended towards a higher expression in male fat tissue at 1.6-fold (p = 0.088).

## Discussion

This study follows on from our recent publication reporting reduced birth weight in rat offspring that had been exposed to a low-dose mixture of pesticides during gestation and lactation^14^. Because of the strong link between fetal exposure to xenobiotics and birth weight, and the fact that low birth weight is a risk factor for developing late-onset diseases both in humans and animals, we wanted to investigate further whether the rat offspring from our study would develop signs of metabolic syndrome in early adulthood.

We observed a lower birth weight in all dose groups and for both sexes when compared to control animals^14^. At pup day 16, the average body weight in the high dose group (Mix-37.5%) was reduced by 15% and 12% in males and females respectively, whereas at 5–6 months the difference appeared reduced to around 5% and was not statistically significant. This suggests some degree of catch-up growth, which potentially could be complete later in adulthood. Since we terminated the study before 6 months, however, the latter question cannot be answered. Nevertheless, catch-up growth in early life to compensate for a low birth weight has been linked to an increased risk of obesity and metabolic disorders^4,13^, meaning that the pesticide-exposed rats in this study could also be at greater risk of developing metabolic disorders. To further address this, we analyzed the offspring with different metabolic parameters in mind.

An oral glucose tolerance test was performed as it offers a dynamic picture of the body’s glucose handling when measuring serum glucose, insulin and glucagon concentrations at different time points. Impaired glucose tolerance will manifest as elevated glucose levels during the latter part of the test period. We did, however, not observe any significant effects in the exposed animals. Notably, we observed a low glucose peak in the glucose test (~8 mmol/l), so a modest impact on the system cannot be completely ruled out. On the other hand, the calculated HOMA-IR score did also not imply any differences between groups, with all animals displaying comparative insulin resistance at 4–5 months of age. Thus, we regard it as unlikely that glucose handling is significantly affected in the young adult rats in this study. But other studies have shown that fetal protein restriction can lead to earlier age-related pre-diabetes despite animals having normal glucose handling^15,16^, so it remains possible that effects could become apparent later, between 1 and 1.5 years of age.

In agreement with our observed lack of effects on both insulin and glucagon plasma levels, we observed no obvious changes in the amount of insulin or glucagon producing cells, nor to liver or muscle tissue, which are tissues involved in glucose metabolism. On the other hand, the weight of the fatty tissue was significantly higher relative to body weight in  pup day 16 males in the high dose group, a tendency persisting into adulthood. These are interesting observations, as they could imply an increase in fat deposition.

Morbid obesity is characterized by a chronic inflammatory state, which is measurable by significant alterations in the expression of inflammation markers caused by a secondary response to altered adipocyte endocrine function. Typically, pro-inflammatory cytokines and chemokines such as TNF-α and IL-6 are up- regulated in obese individuals, whereas anti-inflammatory mediators such as adiponectin are typically down-regulated. Obese individuals will also often display increased insulin levels and insulin-like growth factor-1 (IGF- 1). These factors can therefore be used as biomarkers for various disease states; however, their utility remains to be further refined alongside characterization of additional biomarkers for early detection of disease risk.

Although the pesticide mixture in our study had a clear and significant effect on body weight, we generally did not detect significant changes to plasma hormone levels, with one exception; leptin. Leptin is produced by fat tissues and secreted proportionally to adipose mass and functions as a central regulator of energy homeostasis^17^. Herein, we found leptin levels to be elevated in female offspring at 5–6 months of age in the highest mixture dose (Mix-37.5%). Interestingly, this sexually dimorphic response to intrauterine exposure to xenobiotics is also seen in humans, where serum leptin levels were negatively associated with birth weight in adolescent girls, but not boys^18^. Conversely, *in utero* exposure to either butylparaben or di-isobutylphthalate lowers leptin and insulin levels in the rat^19^, albeit the latter was measured in the fetal compartment and thus not directly comparable to postnatal measurements. Nevertheless, leptin holds some promise as a biomarker, but must be assessed within a temporal- and sex-dependent context. Also, many forms of obesity are characterized by an increased level of circulating leptin at the same time as many obese individuals are in fact resistant to leptin. This suggests that leptin is chiefly a marker of nutrition and not a *bona fide* satiety hormone as first believed, further evidenced by the fact that leptin is incapable of reducing weight in obese individuals and that leptin levels decreases with calorie restriction and weight loss^20^. Finally, these results points to species differences with regards to leptin levels in lean versus obese individuals, so that results may not be directly transferable.

Our morphometric and hormone measurements did not result in clear targets to establish a molecular link between low birth weight and later-life metabolic diseases, or pinpointing any putative biomarkers. But we also performed a targeted gene expression array on adipose tissue. A handful of genes were dysregulated in young adult rats following *in utero* and lactational exposure to the pesticide mixture. For instance, Neuromedin B receptor (*Nmbr*) was up-regulated in all dose groups, whereas its ligand, Neuromedin B (*Nmb*) was unaffected or down-regulated (in Mix-16% group). *Nmb* is expressed in many organs and is involved in energy balance regulation^21,22^. *Nmbr* is also broadly expressed, but receptor function appears at least partially redundant as female knockout mice do not display any severe phenotypes^23^. However, female *Nmbr-*knockout mice are partially resistant to diet-induced obesity. Interestingly, although follow-up expression analyses on a larger biological sample size failed to show a statistically significant alteration in *Nmbr* expression in the exposed animals, we saw a significantly higher (2.5-fold) expression of *Nmbr* in female adipose tissue than in male. Unfortunately, Paula *et al*. (2010) only analysed female knockout mice, whereas it would be potentially insightful to see if there were other effects on male *Nmbr*-knockout mice. The fact is, fat tissues are sexually dimorphic at the molecular level, which highlights a need to consider sex when assessing parameters potentially involving adipocyte tissues, being it centrally or peripherally. To this, we also observed a 1.6-fold higher Leptin (*Lep*) expression in control male adipose tissue compared to female tissue. Although not statistically significant, the trend was persistent across all exposure groups. Thus sex must be considered, not only if using leptin as a biomarker for disease-risk, but also potentially when studying (or treating) obesity-related disorders.

## Concluding Remarks

In our initial study we showed that a mixture of six pesticides, individually well below NOAEL levels, could cause *in utero*-exposed rats to be born with low birth weights^14^. Here, we followed up on this study to see if the offspring also showed signs of developing metabolic diseases in early adulthood. Although we found subtle changes to some key factors, including leptin and glucagon levels in the blood, the study did not reveal strong evidence for heightened risk of metabolic disease or a propensity for weight gain, or any strong new candidates to serve as biomarkers. It is possible that we could have found stronger evidence if animals were also analyzed at later stages and not only as young adults at 5–6 months of age. Unfortunately, this was not feasible for this study, but should be taken into consideration in future studies. Also, it is important to factor in sex when performing analysis in metabolic parameters, as we clearly saw differences between males and females for key regulatory factors. Finally, the fact that we observed some effects at the molecular level that can be related to the development of metabolic diseases following early-life exposure to a mixture of six pesticides at individual levels below NOAEL warrants further studies.

## Materials and Methods

### Chemicals and dose selection

The pesticide mixture included six chemicals and was prepared at three different doses (Table 2). The mixture doses were selected based on 5% effect doses for the single pesticides (a dose estimated to result in a 5% decrease in birth weight), corresponding to a 5% benchmark dose (BMD_5_). A second assumption was that the six pesticides would cause dose addition (DA) responses in a mixture, such that the final mixture of six pesticides individually predicted to reduce birth weight by 5% was denoted Mix-100%; a dose that was not tested as we would expect such a dose to cause maternal toxicity. Rather, the doses used were 5%, 16% and 37% of the calculated Mix-100%. Full details on the rationale and estimation calculations are published in^14^. The six pesticides were purchased from Sigma-Aldrich/Fluka, (Brøndby, Denmark) and were Cyromazine (97%) CAS: 66215-27-8, MCPB (99.8%) CAS: 94-81-5, Pirimicarb (98.7%) CAS: 23103-98-2, Quinoclamine (99.9%) CAS: 2797-51-5, Thiram (99.9%) CAS: 137-26-8, Ziram (98.2%) CAS: 137-30-4. Corn oil (Sigma-Aldrich, Brøndby, Denmark) was used as vehicle control.

**Table 2:** Pesticide mixture composition. The mixture included six pesticides and was prepared at three different doses, denoted Mix-5%, Mix-16% and Mix-37.5%. These mixtures correspond to a total dose of 28, 104 and 210 mg/kg/day, respectively, as derived from a maximum mixture dose of 100%.

Pesticide (purity in %)	CAS No.	Mix-5% (mg/kg bw/day)	Mix-16% (mg/kg bw/day)	Mix-37.5% (mg/kg bw/day)
Cyromazine (97%)	66215-27-8	17.5	64,8	131.25
MCPB (99.8%)	94-81-5	5.0	18.5	37.50
Pirimicarb (98.7%)	23103-98-2	3.0	11.1	22.50
Quinoclamine (99.9%)	2797-51-5	0.5	1.9	3.75
Thiram (99.9%)	137-26-8	1.0	3.7	7.50
Ziram (98.2%)	137-30-4	1.0	3.7	7.50
**Total mixture**		**28**	**104**	**210**

### Animals and exposure

Wistar rats (HanTac:WH) were purchased from Taconic Europe (Ejby, Denmark). Animal experiments were conducted at the DTU National Food Institute’s facilities (Mørkhøj, Denmark) and approved by the Danish Animal Experiments Inspectorate (Ethics authorization number 2012-15-2934-00089 C4) and overseen by the National Food Institute’s Animal Welfare Committee. All methods in this study were performed in accordance with relevant guidelines and regulations. Rats had *ad libitum* access to a complete rodent diet: soy- and alfalfa-free ALTROMIN 1314 (GmbH, Lage, Germany) and acidified tap water. A detailed description of the animal experiments is provided in^14^. Briefly, four groups of 22 time-mated nulliparous pregnant rats each received daily doses of either corn oil (control group) or pesticide mixtures (Table 2; Mix-5%, Mix-16% or Mix-37.5%) from gestation day 7 to pup day 16. Doses were administered to the dams by gavage.

### Offspring body weight after weaning

Offspring were weighed at weaning, at sexual maturation and approximately every 14 days from postnatal day 44 onwards, ending at 5–6 months of age. Specific growth rate (SGR) was defined as weight gain between two points in time divided by previous bodyweight^24^, and calculated for 7 days starting at 14 weeks of age, where also feed consumption was measured.

### Food consumption

Offspring and chow were weighed at 14 weeks, and again 7 days later at 15 weeks. Since animals were housed pairwise, registered food consumption represents the combined consumption of the 2 animals housed together (n = 8–9). In a few of the cages the 2 cohabitant rats were from the same litter (control group: 1 male and 2 female cages; Mix-37.5% group: 2 male and 1 female cages). This potential litter effect was not accounted for in the overall statistical analysis, since a significant litter effect would not be expected for feed consumption in 14 weeks old offspring. Energy efficiency was determined as weight-gain between two time-points divided by energy intake between the two points^24^. Food consumption was expressed as food intake per day per 100 g rat.

### Glucose tolerance test

15 weeks old animals were fasted overnight for 18 h (predominantly light-period) prior to testing. The animals were placed individually in clean cages with bedding and acclimatized for 30 min before test start. Tongue blood was drawn after 15 min and used for measuring fasting insulin levels. At time 0 min, the animals were dosed by oral gavage with 2 g D-glucose/kg bw in the form of an aqueous solution of 500 mg/ml D-glucose. Blood glucose from the tail tip was measured at times −30, 0, 15, 30, 60, 90, and 180 min from time of glucose dosing. Measurements were performed with Accu-Chek Aviva Nano Blood Glucose Monitor system (60308052812) and test strips (06453970016) from Roche, Roche Diagnostics GmbH, Mannheim, Germany. Area-under-curve (AUC) baseline corrected to fasting blood glucose was calculated and compared. A homeostatic model assessment (HOMA) for insulin resistance (IR) was performed using the formula HOMA-IR = (fasting insulin (ng/ml) x fasting glucose (mM))/(22.5 × 0.0417)^25^.

### Necropsy

One male and one female offspring per litter were randomly selected at pup day 16. Rats were weighed, then decapitated under CO_2_/O_2_ anesthesia and blood collected directly into heparinized tubes. Retroperitoneal fat pad, femoral muscle (musculus biceps femoris) and liver were excised and weighed. A piece from one liver lobe was placed in RNAlater and two standard sections fixed in 4% formalin. Similarly, pancreatic tissue was stored at −80 °C in RNAlater or fixed in 4% formalin. At 5–6 months of age, the retroperitoneal fat pad and liver from one male and one female offspring per litter were excised and weighed. Fat pads were stored at −80 °C in RNAlater. Liver sections were fixed in 4% formalin. Pancreatic tissues were excised from both males and females and either stored at −80 °C in RNAlater or fixed in 4% formalin.

### Histology and immunohistochemistry

Paraffin-embedded tissues were sectioned at 3 µm and stained for histopathologic evaluation. One liver section from one female and one male per litter from the control and high-dose groups, both from pup day16 and adulthood, were stained with haematoxylin and eosin (H&E) and evaluated for hypertrophy, vacuolation (macro- and microvesicular) and signs of presence of glycogen (rarefaction) in hepatocytes. An estimation of relative area in the pancreas occupied by β-cells (insulin positive) and α-cells (glucagon positive) was made by point-counting immunohistochemically stained pancreas. β- and α-cells were identified by immunodetection on one section each per animal using rabbit anti-insulin (1:400 dilution, sc-9168, Santa Cruz Biotechnology) and rabbit anti-glucagon (1:500 dilution, sc-13091, Santa Cruz Biotechnology) antibodies, respectively. Sections were weakly counterstained with H&E to differentiate structures in the exocrine pancreas. Point counts of β-cells, α-cells and other pancreatic structures were performed in a grid of 384 points in a minimum of 7 fields per animal for 16 days old offspring (20x magnification) and in a minimum of 10 fields per animal in adult offspring (10x magnification). An average of 64 (SD = 41) and 37 (SD = 19) insulin-positive, and 31 (SD = 24) and 13 (SD = 7) glucagon-positive points per animal were counted in 16 days old and adult offspring, respectively. An average ranging from 2824 to 3762 points representing the total pancreatic tissue was counted per animal. Ratios between i) β-cell area and area of total pancreatic tissue, ii) α-cell area and area of total pancreas tissue, and iii) β-cell area and α-cell area were calculated. The point counting on pancreas sections was performed in Image Pro Plus 7.0 software (Media Cybernetics, Bethesda, MD, USA). All assessments of histological changes (livers and pancreas) were performed blinded to treatment groups.

### Measurements of metabolic biomarkers in plasma

Plasma levels of several metabolic hormones were measured using a MILLIPLEX® MAP Rat Metabolic Bead panel kit (#RMHMAG-84K) from Millipore, (Billerica, MA, USA). The kit allows for simultaneous quantification of C-peptide 2, GIP (Gastric Inhibitory Polypeptide), PYY (Pancreatic peptide YY) IL-6, Insulin, Leptin, and MCP-1. All analyses were performed according to the manufacturer’s instructions, using a Luminex IS 100® platform (Luminex Corporation, Austin, TX, USA). Acquired fluorescence data were analyzed using the Bioplex v 5.0 software (Bio-rad Laboratories; USA). Plasma levels of insulin and glucagon were analyzed using two separate ELISA kits from Mercodia (Rat Insulin article no. 10-1250-01 and Glucagon article no. 10-1281-01) according to the manufacturer’s protocol. Absorbance was measured using an Enspire® Multilable Plate Reader (PerkinElmer).

### Rat gene array

The Rat Obesity RT2 Profiler^TM^ PCR Array (Qiagen; PARN-017ZA) was used to profile the expression of 84 genes simultaneously. Three retroperitoneal fat pad samples (2x male, 1x female) were randomly selected from each group: Control, Mix-5%, Mix-16% and Mix-37.5%. Total RNA was extracted from tissues stored in RNA-later (Life Technologies, Europe BV, Denmark) using the RNeasy Lipid Tissue Mini kit (Qiagen) including on-column DNase I treatment as per manufacturer’s instructions. cDNA was synthesised from 1 µg total RNA using the recommended RT2 First Strand Kit as described by the manufacturer (Qiagen). Samples were prepared for 384-well format and run on a 7900HT Fast Real-Time PCR System (Applied Biosystems). Relative transcript levels were determined by the comparative Ct-method using the Qiagen on-line Data Analysis Center.

### RT-qPCR analysis

The protocols were essentially as described previously^26^, with 500 ng total RNA used to synthesise cDNA. TaqMan Gene Expression Assays (Life Technologies) were: *Agrp* (Rn01431703), *Il1b* (Rn00580432), *Lep* (Rn00565158), *Lepr* (Rn01433205), *Nmb* (Rn01478123), *Nmbr* (Rn00680847), *Npy* (Rn01410145), *Npy1r* (Rn02769337), *Pparg* (Rn00440945), *Ppargc1a* (Rn00580241) and *Tnf* (Rn99999017). RT-qPCR experiments were run on a 7900HT Fast Real-Time PCR System (Applied Biosystems) in a 384-well format using 3 µl diluted (1:20) cDNA per reaction. Relative transcript abundance was calculated by the comparative Ct-method with the geometric mean of the reference genes *Hprt* (Rn01527840) and *Rpl13a* (Rn00821946).

### Statistics

For all analyses, the alpha level was set at 0.05 and the litter was the statistical unit. Statistical analysis of metabolic biomarker data was performed using Sigma Plot v.11.0 (Systat Software Inc.). Data were tested for normality and homogeneity of variance. Data were analyzed by one-way analyses of variance (ANOVA) and, if significant followed by Dunnett’s post hoc test. Body weights over time were analyzed by repeated measures in a mixed model ANOVA. Absolute organ weights were analyzed by one-way analyses of variance (ANOVA) with body weight as a covariate, followed by Dunnett’s post hoc test of LSMeans.

Liver histological scoring data were analyzed using a Fisher’s exact test (2 × 2). Pancreas point counting data were analyzed using a two-tailed t-test, as only control and high-dose groups were investigated histologically. RT-qPCR data were analyzed by one-way ANOVA across exposure groups, and two-tailed unpaired t-test for comparison between male and female controls only.

## Electronic supplementary material

Supplementary Figure 1

Dataset 1
