# Supplementary material for: Effects on metabolic parameters in young rats born with low birth weight after exposure to a mixture of pesticides
Source: Sci Rep. 2018 Jan 10;8:305. doi: 10.1038/s41598-017-18626-x (PMC5762645; doi:10.1038/s41598-017-18626-x)
Supplement: Supplementary file 1 — Supplementary Figure 1 [file 41598_2017_18626_MOESM1_ESM.pdf]

Supplementary files:

Effects on metabolic parameters in young rats born with low birth weight after exposure to a mixture of pesticides

Terje Svingen*, Louise Ramhøj, Karen Mandrup, Sofie Christiansen, Marta Axelstad, Anne Marie Vinggaard & Ulla Hass

Division of Diet, Disease Prevention and Toxicology, National Food Institute, Technical University of Denmark, Kemitorvet Bldg. 202, Kgs. Lyngby DK-2800, Denmark.

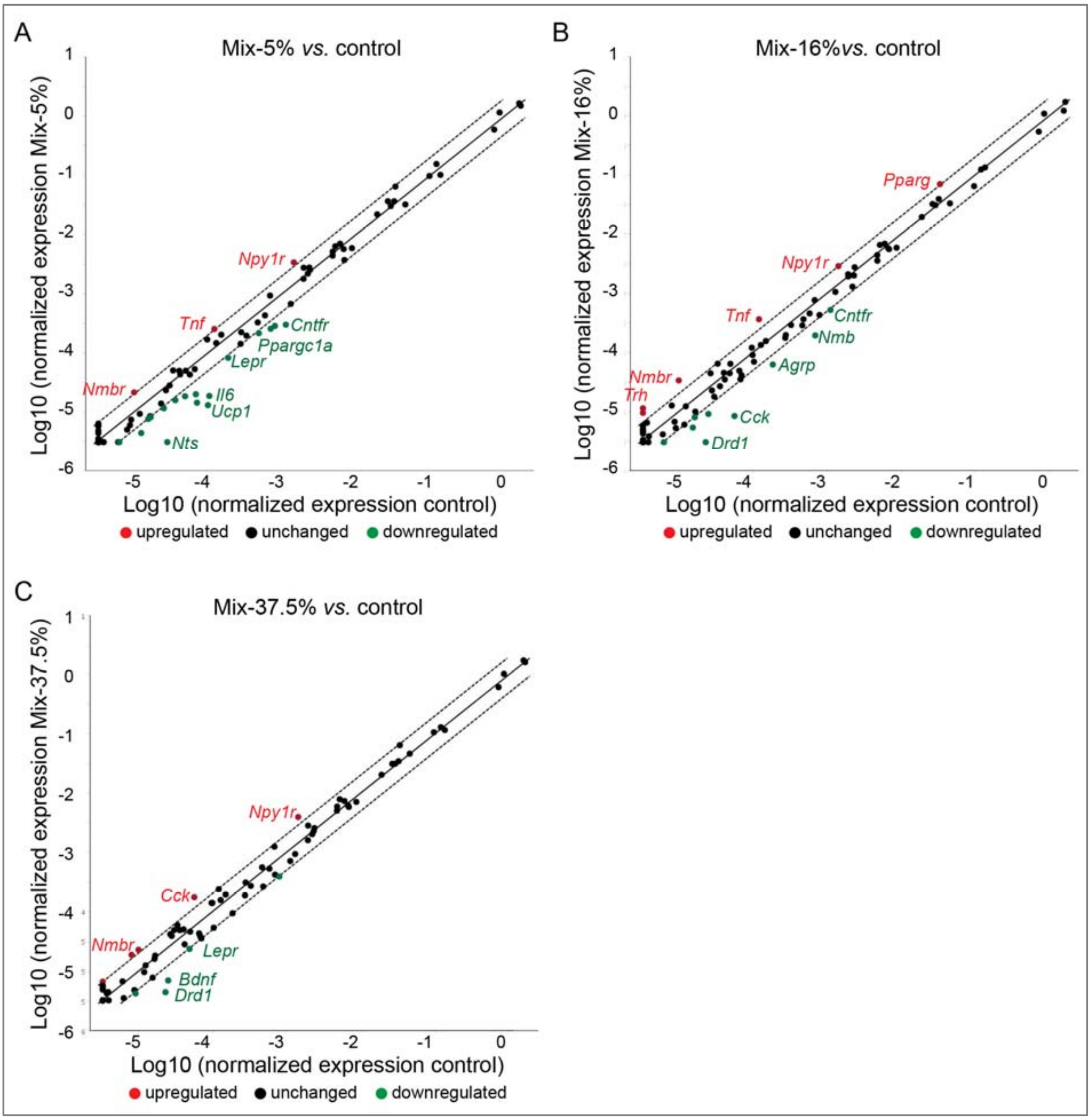

Suppl. Fig. 1: Obesity gene array on fat tissue from rats after exposure to a mixture of pesticides.

Scatter-plots of differentially expressed obesity-related genes in retroperitoneal fat pads from control versus exposure groups: A) Mix-5%, B) Mix-16%, and C) Mix-37.5%. Relative fold expression was determined from average transcript abundance across groups (N=3 from each group). Dotted lines depicts 2-fold regulation relative to Control Mean.
